# Insanity and Research

**Published:** 1921-09-03

**Authors:** 


### INSANITY AND RESEARCH.

It is a common reproach, addressed chiefly to those medical men who work in asylums, that our knowledge of the causation of insanity makes such slow progress towards accuracy. Those who assert this are usually ignorant of what has already been done, or they are annoyed because their own conceptions as to causation are not received with enthusiasm. Insanity is regarded by many as something like the French Republic—one and indivisible, and as, therefore, possibly caused by one factor, such, for example, micro-organisms. Even this is looked upon as a wonderful and new suggestion, although it has been long recognised as one of the causes, and researches of Noguchi and others have demonstrated, apparently beyond any doubt, that that fell disease, general paralysis of the insane, is due to the action of the spirochæte.

Much useful work has been done already—and a considerable amount of it in this country—in spite of the fact that little encouragement or, comparatively, small financial aid have been given. There has been a vast deal of fuss—much of it unnecessary—about the state of asylums in this country; but how little has been said about the situation which appears to be arising in regard to a very important institution—the Maudsley Hospital! It will be recalled that the late Dr. Maudsley gave a large sum of money for the erection of a hospital for the study of mental disorders and their treatment. The hospital was built, and was used for soldier patients. In addition, a section of the hospital was given up to laboratories, and these, under the direction of Sir Frederick Mott, have produced a large and valuable output of work. Details of this were given by Sir Frederick in his second Maudsley Lecture. He gathered round him a band of enthusiastic workers, and the fame of the Maudsley Hospital had already begun to spread. His own researches, especially in regard to the etiology of *dementia præcox*, were alone sufficient to justify the existence of a laboratory. In addition to the research work, there has grown up quite a school, to which students, British and foreign, have been attracted, and systematic courses of instruction have been carried on.

It is suggested that all this is going to be changed, that only routine examination of specimens will be made, and that research will cease. Apparently this is to be done on the grounds of expense, and, if so, it is only another aspect of the short-sighted policy that is still the rule in this country. England dislikes spending money on prophylatic measures. It is to be hoped that there will be a reconsideration of the whole matter. Surely, in view of the recent clamour, this is an expense which might easily be justified to the ratepayers. True, pathological research is not specially picturesque and cannot give rise to waves of sentiment on its behalf. Yet if only half the money that is being spent on the printing and publishing of the volumes given up to the flatulent discussion of so-called psychological subjects were devoted to this, which is of practical value, the problem of research at the Maudsley Hospital would be solved. But it is easier to spend money on advertisements!

